# Arrested neural and advanced mesenchymal differentiation of glioblastoma cells-comparative study with neural progenitors

**DOI:** 10.1186/1471-2407-9-54

**Published:** 2009-02-14

**Authors:** Piotr Rieske, Ewa Golanska, Magdalena Zakrzewska, Sylwester Piaskowski, Krystyna Hulas-Bigoszewska, Magdalena Wolańczyk, Malgorzata Szybka, Monika Witusik-Perkowska, Dariusz J Jaskolski, Krzysztof Zakrzewski, Wojciech Biernat, Barbara Krynska, Pawel P Liberski

**Affiliations:** 1 Department of Molecular Pathology and Neuropathology, Chair of Oncology, Medical University of Lodz, Czechoslowacka 8/10, 92-216 Lodz, Poland; 2 Department of Oncological Pathology, Chair of Oncology, Medical University of Lodz, Paderewskiego 4, 93-509 Lodz, Poland; 3 Department of Neurosurgery, Medical University of Lodz, Kopcinskiego 22, 90-153 Lodz, Poland; 4 Department of Neurosurgery, Polish Mother Memorial Hospital Research Institute Lodz, Rzgowska 281/289, 93-338 Lodz, Poland; 5 Department of Neuropathology and Molecular Pathology, Medical University of Gdañsk, Dêbinki 7, 80-211 Gdañsk, Poland; 6 Department of Neurology, Temple University School of Medicine, 3401 N Broad St. 558 Parkinson Pavilion, Philadelphia, PA 19140, USA

## Abstract

**Background:**

Although features of variable differentiation in glioblastoma cell cultures have been reported, a comparative analysis of differentiation properties of normal neural GFAP positive progenitors, and those shown by glioblastoma cells, has not been performed.

**Methods:**

Following methods were used to compare glioblastoma cells and GFAP+NNP (NHA): exposure to neural differentiation medium, exposure to adipogenic and osteogenic medium, western blot analysis, immunocytochemistry, single cell assay, BrdU incorporation assay. To characterize glioblastoma cells *EGFR *amplification analysis, LOH/MSI analysis, and *P53 *nucleotide sequence analysis were performed.

**Results:**

*In vitro *differentiation of cancer cells derived from eight glioblastomas was compared with GFAP-positive normal neural progenitors (GFAP+NNP). Prior to exposure to differentiation medium, both types of cells showed similar multilineage phenotype (CD44+/MAP2+/GFAP+/Vimentin+/Beta III-tubulin+/Fibronectin+) and were positive for SOX-2 and Nestin. In contrast to GFAP+NNP, an efficient differentiation arrest was observed in all cell lines isolated from glioblastomas. Nevertheless, a subpopulation of cells isolated from four glioblastomas differentiated after serum-starvation with varying efficiency into derivatives indistinguishable from the neural derivatives of GFAP+NNP. Moreover, the cells derived from a majority of glioblastomas (7 out of 8), as well as GFAP+NNP, showed features of mesenchymal differentiation when exposed to medium with serum.

**Conclusion:**

Our results showed that stable co-expression of multilineage markers by glioblastoma cells resulted from differentiation arrest. According to our data up to 95% of glioblastoma cells can present *in vitro *multilineage phenotype. The mesenchymal differentiation of glioblastoma cells is advanced and similar to mesenchymal differentiation of normal neural progenitors GFAP+NNP.

## Background

The biology and clinical prognosis of glioblastoma is a subject of intense research, and several reports on differentiation pathways of glioblastoma cells isolated from high-grade gliomas have been recently published [1,2]. In addition to neuronal or glial lineages, mesenchymal differentiation has recently been described in cell cultures obtained from some of these tumors [3,4]. Moreover, a co-expression of markers typically identified either in glial or neuronal lineages, has been previously described in glioblastoma cells [2]. It was suggested that this phenotype may be a consequence of dedifferentiation/transdifferentiation of transformed cells [2]. Although features of variable differentiation in glioblastoma cell cultures have been reported, a direct comparison of differentiation properties of normal neural cells exhibiting multilineage phenotype, and those shown by glioblastoma cells, has not been performed.

Previously we have shown that normal GFAP positive cells with characteristics of normal neural progenitors (GFAP+NNP) co-express neuronal, glial, and mesenchymal markers and differentiate into neuronal, glial, and non-neural cells [5-7]. Our recent studies showed mesenchymal differentiation of these cells [8].

The recent demonstration that tumor cells isolated from some human gliomas can differentiate into neural and mesenchymal derivatives, and our earlier observations of neural and mesenchymal differentiation of GFAP+NNP cells, with multilineage (discordant) phenotype, inspired us to compare *in vitro *inducible differentiation and phenotypic changes of GFAP+NNP and glioblastoma cells.

The similarities between GFAP+NNP cells and a subpopulation of glioblastoma cells isolated from high-grade gliomas presented in this study shed new light on glioblastoma biology.

## Methods

### GFAP+NNP growth and differentiation

GFAP+NNP, isolated from the cerebrum of human fetuses, were purchased from Lonza, formerly Cambrex (CC-2565, NHA-Normal Human Astrocytes; Walkersville, MD). GFAP+NNP at passage 0 (or rarely passage 1) were grown for 8 hours in expansion medium (supplemented with rhEGF, insulin, AA with 3% fetal bovine serum: AGM Bullet Kit Media, Lonza), then for 24 hours in serum-starvation medium; DMEM/F12 medium (Gibco) supplemented with N2 (10×) (Gibco), insulin (10 ng/mL; Invitrogen), and epidermal growth factor (EGF) (10 ng/mL; Invitrogen). Then, the medium was changed to neural differentiation medium: DMEM/F12 supplemented with N2 (10×). The cells were grown in the differentiation medium for 2–20 days.

### Glioblastoma aggregate formation

Tissue samples were obtained from patients with glioblastoma treated in the Department of Neurosurgery, Polish Mother Memorial Hospital Research Institute of Lodz and Department of Neurosurgery Medical University of Lodz, Poland. All samples were collected under protocols approved by Medical University of Lodz. The tumor cells were dispersed by means of collagenase type IV (20 U/mL, 37°C). Subsequently, the cells were for 12 hours in expansion medium. Twelve hours later, the medium was changed to the serum-starvation medium, and aggregates were isolated after 1–4 days of incubation. For each tumor 20–40 aggregates were tested.

### Glioblastoma aggregate propagation and characterization

The aggregates were isolated and transferred into cell culture dishes covered with Matrigel (Growth Factor-reduced; BD Discovery Labware, Bedford, MA) and cultured in neural differentiation medium: DMEM/F12 supplemented with N2 (10×). After 12–24 hours of incubation, it was observed that cells were released from the aggregates. The aggregates were then gently removed by means of a 1-mL pipette and the cells which migrated out of the aggregates were left on the dish for further experiments. The aggregate-derived cells were immunocytochemically stained after 12–24 hours and at 5, 10, 15, and 20 days of growth.

The aggregates could be propagated for at least 10 months, incubated in neural differentiation medium and transferred every 5–20 days. The experiments presented in this paper were performed after three to six transfers of the aggregates into new medium (no longer than five weeks of propagation).

### Exposure of glioblastoma cells to medium with serum

Glioblastoma cells forming aggregates, non-selected glioblastoma cells and undifferentiated GFAP+NNP were cultured in alpha-MEM media containing 10% FBS (fetal bovine serum).

### Western blot analysis

The protein extracts from cell cultures were obtained with the use of NucleoSpin (Macherey-Nagel). The commercially available Brain Tissue Lysate was used as a positive control in Western blot analysis (Abcam). Equal amounts of protein extracts, 25 μg per line, were separated by 5% or 10% SDS-PAGE followed by transfer onto Immobilon™-P polyvinylidene difluoride membranes (Sigma-Aldrich). After blocking of nonspecific binding, the membranes were incubated for 1 hour at room temperature with primary antibodies (Table 1). After extensive washing in TBST buffer, the membranes were incubated for 1 hour at room temperature with species-specific secondary antibody (Santa Cruz Biotechnology, Inc.), diluted 1:2000. Membranes were then washed in TBST buffer and finally in TBS buffer (TRIS Buffered Saline; pH 7.5); antigen-antibody complexes were detected by enhanced chemiluminescence, using Chemiluminescence Luminol Reagent (Santa Cruz Biotechnology, Inc.) and visualized with the use of BIO-RAD camera and Quantity One software.

**Table 1:** Primary antibodies used for Western blot (WB) and immunocytochemical staining (IC)

I Ab	Host	Manufacturer	Application (Dilution)
anti-nestin	mouse	Santa Cruz Biotechnology, Inc.; sc-23927	WB (1:200); IC (1:100)

anti-GFAP	mouse	Chemicon; MAB360	WB (1:800); IC (1:400)

anti-CD44	mouse	Santa Cruz Biotechnology, Inc.; sc-7297	WB (1:100); IC (1:100)

anti-MAP-2	rabbit	Santa Cruz Biotechnology, Inc.; sc-20172	WB (1:200); IC (1:100)

anti-nestin	rabbit	Santa Cruz Biotechnology, Inc.; sc-20978	IC (1:100)

anti-CD133	rabbit	Santa Cruz Biotechnology, Inc.;sc-30220	IC (1:100)

anti-βIII-tubulin	rabbit	Sigma; T 2200	WB (1:250); IC (1:250)

anti-Fibronectin	rabbit	Sigma; F 3648	IC (1:200)

anti-SOX2	rabbit	Chemicon; AB5603	IC (1:1000)

anti-GFAP	goat	Santa Cruz Biotechnology, Inc.; sc-6171	IC (1:50)

anti-vimentin	goat	Chemicon; AB-1620	WB (1:100); IC (1:40)

anti-βIII-tubulin	mouse	Chemicon MAB 1637	IC (1:200)

anti-TH	mouse	Santa Cruz Biotechnology, Inc.; sc-25269	IC (1:100)

anti-BrdU	mouse	Sigma B 8434	IC (1:500)

### Immunocytochemistry

Immunocytochemistry assays for double- or triple-immunofluorescent labeling were performed. For immunofluorescence studies, cells were grown on tissue-culture chamber slides or for single cell assay experiment, in 16-well chamber slides (Nunc). The cells were fixed with 4% paraformaldehyde for 15 minutes, permeabilized with 0.1% Triton X-100 for 10 minutes at room temperature and blocked with 2% donkey serum in PBS for 1 hour at room temperature. For double or triple immunolabeling, fixed cells were subsequently incubated with appropriate primary antibodies (Table 1) for 1 hour at room temperature. Double- or triple-labeling was achieved by simultaneous incubation with a combination of species-specific fluorochrome-conjugated secondary antibodies (1 hour, room temperature). For double immunolabeling, a mixture of donkey anti-rabbit AlexaFluor^®^488 (dilution 1:250) and donkey anti-mouse AlexaFluor^®^594 (dilution 1:250) antibodies (Molecular Probes) were applied. For triple labeling, the following combination of antibodies was used: donkey anti-rabbit AlexaFluor^®^488 (dilution 1:250), donkey anti-mouse AlexaFluor^®^594 (dilution 1:250), donkey anti-goat AlexaFluor^®^350 (dilution 1:250); Molecular Probes. After a final rinse with PBS, the slides were mounted using ProLong^® ^Gold Antifade Reagent (Molecular Probes). For nuclei staining, the ProLong^® ^Gold Antifade Reagent with DAPI (Molecular Probes) was used. The slides were coverslipped and examined using an Olympus BX-41 fluorescence microscope. Semi-quantitative analysis based on measurement of fluorescence intensity was performed with the use of WCIF Image J software (Wright Cell Imaging Facility, Toronto Western Research Institute). MAP2+^high ^signal was defined based on current measurements and results published by Witusik et al [6]. Cells showing intensity higher than 120 units/pixel were defined as MAP2+ ^high^. For immunocytochemical BrdU staining, the vendor protocol was applied (Sigma).

### *EGFR *amplification analysis

Multiplex PCR was performed for evaluation of *EGFR *amplification with superoxide dismutase 1 (*SOD1*) used as a reference gene. *EGFR *and *SOD1 *were amplified using the following primers: 5'-ctactagaagttgatggctt-3' and 5'-ggtccatgaaaaagcagatg-3' (110 bp); 5'-ttaagaagacttggtggtccatgaaaaagcagatg-3' and 5'-aaaaaagcttggaatgtttattgggcgatcc-3' (163 bp). PCR products were separated by electrophoresis in 2% agarose gel, visualized using a Bio-Rad Gel Doc 1000 and analyzed with Molecular Analyst software as described before [9].

### LOH and MSI analysis

DNA was isolated from the cells obtained from the aggregates and from immunostained cells, original tumor cultures and blood isolated from the patients, by means of Macherey-Nagel DNA/RNA/Protein purification kit. LOH and MSI analyses were performed using paired tumor specimens and corresponding peripheral blood samples. The following LOH markers were used: D1S2734, D1S197, D1S162 D1S156, D9S319, D9S162, D10S587, D10S1267, D17S1828. MSI markers have already been described [10]. Forward primers were 5'-end fluorescence-labeled. PCR was performed in thermocycling conditions individually established for each pair of primers. PCR products were denatured and gel electrophoresis in LiCor automatic sequencer system was applied to the separation and analysis of PCR-generated alleles.

### Nucleotide sequence analysis of *P53*

Four genomic regions of *P53 *gene (exons 5–8) were amplified by PCR using sets of primers encompassing each exon [11]. Sequencing was performed as described before [11] using the dideoxy termination method, SequiTherm Excel DNA Sequencing Kit (Epicentre Technologies) and LiCor automated sequencer.

### BrdU incorporation assay

To assess the mitotic activity of glioblastoma cells, 10 μM BrdU (Sigma, St. Louis, MO), a marker of DNA synthesis, was added to the cells cultured in expansion media for 48–72 hours. To assess the mitotic activity of cells derived from glioblastomas, 10 μM BrdU was added to DMEM/F12 media supplemented with N2 on days 1 and 15 of culture. Similarly, BrdU was added to the glioblastoma cells passaged in 10% alpha-MEM. BrdU incorporation was assessed after 5, 10, 15 and 20 passages. After 48–72 hours of incorporation, the cultures were fixed with 4% paraformaldehyde for 15 minutes and processed for immunocytochemistry.

### Exposure of glioblastoma cells to the adipogenic differentiation medium

Glioblastoma cells showing CD44+, Vimentin+, Fibronectin+, and neural markers positive, phenotype were plated in 6-well tissue culture dishes, and allowed to reach 80% confluence. Triplicate wells were incubated for 3 weeks in adipogenic medium, containing 0.5 μM hydrocortisone (Sigma), 0.5 mM isobutylmethylxanthine (IBMX) (Sigma) and 60 μM indomethacin (Sigma). The medium was replaced every 3 to 4 days. To assess lipid deposition, the cultured cells were washed with PBS and fixed for 1 hour with 10% formalin, then stained for 15 minutes with a diluted Oil Red-O solution (Sigma) with shaking at room temperature, then washed three times with dH_2_O, and subsequently examined under a light microscope. Negative controls were glioblastoma cells showing mesenchymal phenotype but not exposed to adipogenic medium; positive controls consisted of marrow stromal cells exposed to adipogenic medium and stained with Oil Red-O.

### Single cell assay

Glioblastoma cells obtained after dislodging the aggregates were used for single cell assay: one cell was suspended in the medium directly into one conical well of a 96-well or 16-well immunocytochemistry plate. Then, the cells were grown in several conditions: serum-starvation medium, neural differentiation medium, and expansion medium, as described under the GFAP+NNP growth and differentiation section. The single cell assay was also started at passage 0, just after the beginning of the tumor cell cultures. The cells were stained, photographed and subsequently DNA was isolated and LOH analysis was performed to check if clones were obtained from the tumor cells.

## Results

### Subpopulation of glioblastoma cells with multilineage phenotype has the ability to form aggregates

**Figure 1:**
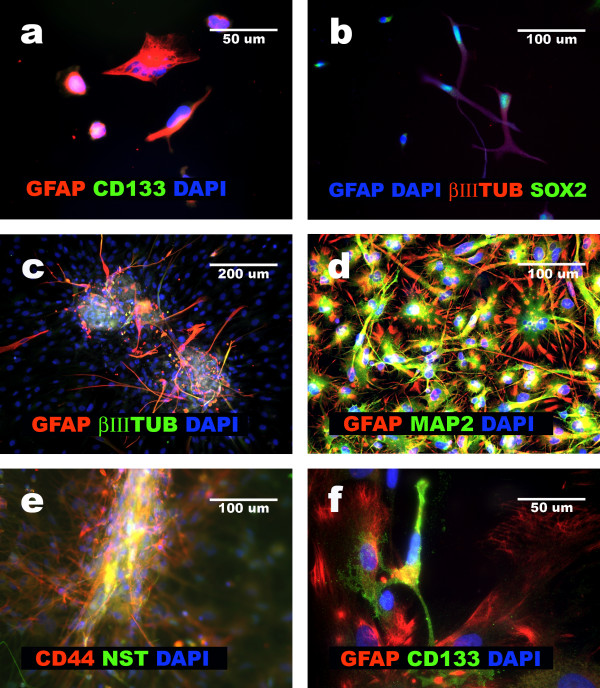
**Characterization of GFAP+NNP and GBM cells showing discordant phenotype**. **a**, GFAP+NNP cells negative for CD133 and GFAP positive; **b**, GBM4 cells positive for GFAP, Beta III-tubulin and SOX-2 and cells SOX-2, Beta III-tubulin and GFAP negative; **c**, GBM3 cells forming aggregate; **d**, GBM1 cells released from aggregate positive for GFAP and MAP2; **e**, aggregated and released from aggregate cells positive for CD44 and Nestin (GBM 3); **f**, GBM2 cells original culture cells positive for CD133, and cells positive for GFAP.

Cells isolated from eight glioblastomas, as well as GFAP+NNP, were grown in the same culture conditions: first in expansion medium, then in serum-starvation medium, and finally in neural differentiation medium, as described in Materials and methods. All undifferentiated GFAP+NNP presented the multilineage phenotype, defined as co-expression of GFAP, CD44, Beta III-tubulin, MAP2 and Nestin, SOX-2, Vimentin [5-7]. GFAP+NNP were CD133 negative (Fig. 1a). In initial monolayer cultures isolated from eight glioblastomas, 10% (GBM6) to 95% (GBM1) of cells presented an evident multilineage phenotype (Table 2). All glioblastoma cells with multilineage phenotype were also SOX-2 positive (Fig. 1b). In addition to the population of cells with multilineage phenotype, cells with one or more of the following phenotypes were also observed in different glioblastoma cultures: MAP2+^high^/GFAP+/CD44-; CD44+/GFAP-/MAP2-; GFAP+/CD44+/MAP2-; MAP2+^high^/GFAP-/CD44-; CD133+/CD44+/MAP2+/GFAP+ and CD133+/MAP2-/GFAP- (Table 2).

**Table 2:** Phenotypic and genetic features of GFAP+NNP and glioblastoma cell cultures

Feature	GBM1	GBM2	GBM3	GBM4	GBM5	GBM6	GBM7	GBM8	NHA
% of multilineage phenotype cells in original culture	95%	70%	63%	45%	30%	10%	15%	45%	100%

Other than multilineage phenotypes observed at the beginning of *in vitro *culture	glial, neuronal-intermediate, mesenchymal	glial, mesenchymal	glial, mesenchymal	glial, mesenchymal	glial, neuronal-intermediate, mesenchymal	glial, mesenchymal	other, not defined	glial, neuronal-intermediate, neuronal, mesenchymal	0%

Clonable	-	+	-	+	-	+	-	-	-

% of CD133+ cells	0%	3%	0%	2%	2%	1%	2%	3%	0%

Aggregate-forming ability	+	+	+	+	-	-	-	+	+

Aggregates stability	+	+	+	+	nt	nt	nt	+	-

Mesenchymal differentiation *	(+) 10	(+) 15	(+) 10	(+) 15	(+) 10	(+) 10	**-**	(+) 10	(+)5

Molecular background	EGFR+, P53wt	EGFR-, P53mut	EGFR+, P53wt	EGFR-, P53wt	EGFR+, P53wt	EGFR-, P53mut	EGFR-, P53 wt	EGFR-, P53mut	EGFR-, P53wt

The phenotypes: multilineage phenotype, in brief CD44+/MAP2/GFAP+ (in detail Beta III tubulin+/Nestin+/Vimentin+/SOX2+); glial, GFAP+/CD44+/MAP2-; neuronal, MAP2+/GFAP-/CD44-; neuronal-intermediate, GFAP+/MAP2+/CD44-; mesenchymal, in brief CD44+/GFAP-/MAP2- (in detail CD44+/Vimentin+/Fibronectin+/GFAP-/MAP2-/Beta III-tubulin-/Nestin-/SOX2-).

EGFR+, amplification of *EGFR *gene; EGFR-, no amplification of *EGFR *gene; P53wt, wild type; P53mut, mutation in P53; nt, not tested.

GBM, glioblastoma

*Number of passages required to present only mesenchymal (non-neural) cells.

Between 24–48 hours of incubation in the serum-starvation medium, glioblastoma cells growing initially in a monolayer, began forming aggregates (Fig. 1c). A spontaneous ability to form aggregates consisting of multilineage tumor cells was observed in five of eight glioblastomas (GBM1, GBM2, GBM3, GBM4, GBM8), which presented more than 40% of cells with multilineage phenotype. The aggregates were very stable and able to be transferred and micro-surgically manipulated. To confirm that the aggregates were formed by a pure population of tumor cells with the multilineage phenotype, every aggregate was gently cut into two parts. One part was removed from the culture for DNA/protein isolation and immunocytochemical staining, and the second part was further propagated.

**Figure 2:**
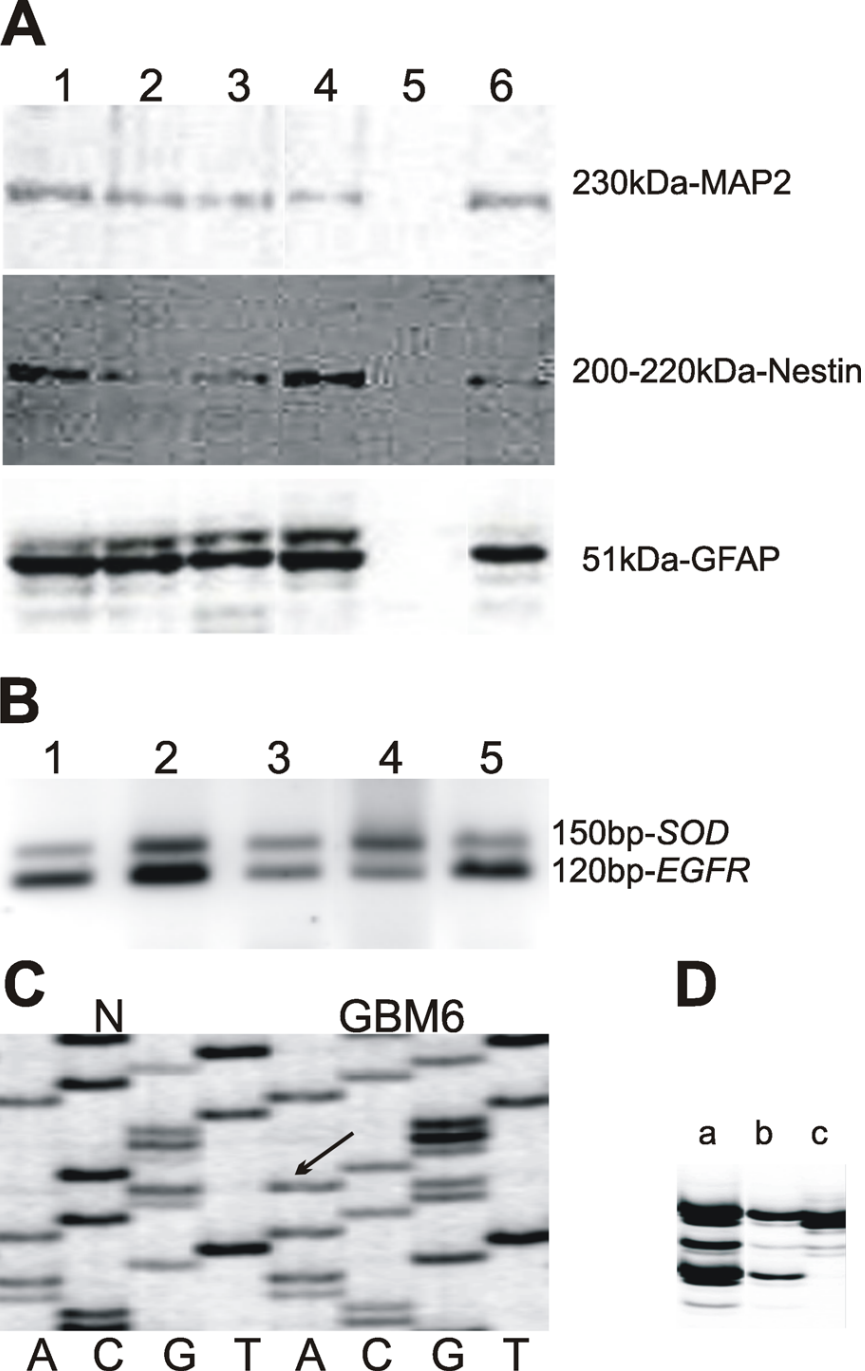
**Molecular characterization of glioblastomas**. **a**, Western blot analysis to show MAP2, Nestin and GFAP expression in cultured GBM1, GBM2, GBM3, GBM4 (lanes 1–4) cells and control brain lane 6; lane 5, negative control blood cells. **b**, Multiplex analysis allowing to detect *EGFR *amplification. Lanes 1, 2, 3 samples showing amplification of *EGFR*; lane 4, negative control; lane 5, positive control. **c**, Sequencing of *P53 *normal sample and sample presenting mutation of *P53 *(GBM6). **d**, LOH analysis. Lane a, blood sample; lane b, tumor sample frozen tissue contaminated with cells presenting ROH (ROH, retention of heterozygosity); lane c, lack of cells with ROH, DNA isolated from aggregate.

Glioblastoma cells forming aggregates, as well as undifferentiated GFAP+NNP, co-expressed SOX-2, Nestin, Beta III-tubulin, MAP2, GFAP, Vimentin, Fibronectin and CD44, when cultured under serum-starvation media. The expression of these markers was detected by immunocytochemistry (Fig. 1a–f) [5-7], and Western blotting (Fig. 2a). Under these conditions, GFAP+NNP did not form aggregates and were maintained in monolayer. Because the aggregates were formed by homogenous tumor cells, in terms of the multilineage phenotype, aggregate formation allowed for separation of these cells from other tumor cells in glioblastoma cultures. Aggregates, but not monolayer of glioblastoma cells, were used for a majority of further experiments.

### Analysis of molecular background of glioblastomas: *EGFR *amplification and *P53 *mutations

The phenotypical discrepancies of eight glioblastomas presented here prompted us to perform basic molecular testing. The molecular alterations in glioblastoma are variable, however, two predominant genetic pathways typical for primary (*EGFR *amplifications) and secondary glioblastomas (mutations of *P53*) have been described [12,13]. Therefore, we performed sequencing of *P53 *gene and *EGFR *amplification analysis by multiplex PCR in DNA isolated directly from the tumors and from the respective cell cultures. *EGFR *amplification was identified in GBM1, GBM3 and GBM5, whereas GBM2, GBM6 and GBM8 showed *P53 *gene mutations (Table 2, Fig. 2b–c). None of these alterations were found in GBM4 and GBM7 (Table 2). The statistical analysis did not reveal any important association between the molecular background and cellular phenotype.

### LOH and MSI analyses show that aggregates obtained from glioblastoma contain only tumor cells

Non-neoplastic stem cells have been previously described to infiltrate tumor tissue; we verified whether the population of the aggregate-forming cells was composed purely of tumor cells. We showed that DNA isolated from frozen tumors was heterogeneous, most likely as a result of contamination by non-tumor cells: both normal DNA and allele with chromosomal loss was found in the tumor samples. However, DNA isolated from the aggregates showed no retention of the normal allele in LOH analysis, indicating that these aggregates consisted of only tumor cells (GBM1-GBM4) (Fig. 2d).

### GFAP+NNP differentiation: presence of neuronal intermediates

**Figure 3:**
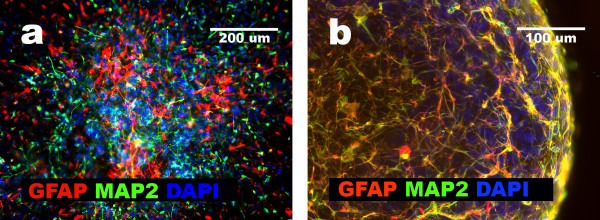
**Comparison of GBM and GFAP+NNP aggregate stability**. **a**, GFAP+NNP aggregate dispersed after attachment to the surface, separated cells positive either for GFAP or MAP2; **b**, GBM4 stable aggregate cells positive for GFAP and MAP2.

**Figure 4:**
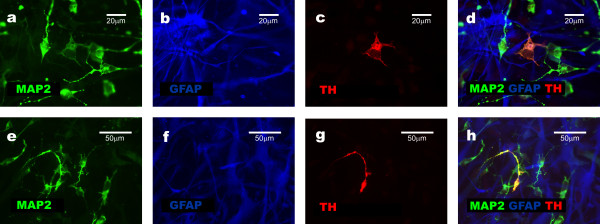
**TH, GFAP and MAP2 expression presented by derivatives of GFAP+NNP**. **a-d**, TH expression observed in the cell showing downregulated GFAP and upregulated MAP2 expression. **e-h**, TH expression in maturating neuronal cells showing high expression of MAP2 and lack of GFAP.

**Figure 5:**
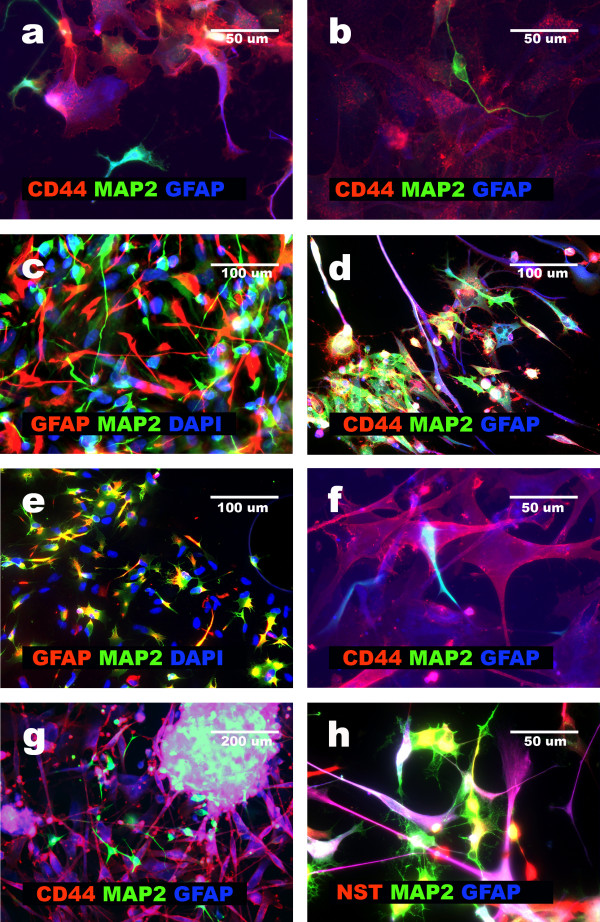
**GFAP+NNP vs. GBM neural differentiation**. **a**, GFAP+NNP cells at passage 1, three days of neural differentiation. Cells positive for CD44 and GFAP, and cells positive for MAP2 and GFAP but negative for CD44. MAP2 dominates but GFAP is visible. Cells CD44+, GFAP+ are also presented. **b**, GFAP+NNP cells at passage 1, four days of neural differentiation. Two types of cells are visible: positive for CD44 and GFAP, and cells MAP2+; GFAP+, CD44-. **c**, GFAP+NNP cells at passage 1, 5 days of neural differentiation: GFAP+ MAP2- and MAP2+ GFAP- cells. **d**, GBM2: CD44, GFAP, MAP2 positive cells- cells sustaining discordant phenotype. **e**, GBM 1 cells released from aggregate, 15 days of neural differentiation: cells GFAP- MAP2- and MAP2+ GFAP+ are visible. **f**, GBM3: CD44 negative, GFAP and MAP2 positive cells in the centre. **g**, GBM8: strongly MAP2 positive cells and GFAP, CD44 positive cells released from the aggregate. **h**, GBM4: GFAP and MAP2 positive cells sustaining Nestin expression.

Next, we replaced the serum-starvation medium with neural differentiation medium in GFAP+NNP cultures as well as glioblastoma cell aggregates. Under these conditions, GFAP+NNP rarely formed aggregates. If aggregates appeared, they were unstable and completely dispersed after one or two transfers due to massive release of differentiating cells. The dispersed cells showed features of differentiation and grew in monolayer (Fig. 3a,b). Using triple immunocytochemical staining, we showed that at early stages of differentiation (after 2–3 days of incubation in the neural differentiation medium), in 25–35% of the GFAP+NNP derivatives, MAP2+^high^/GFAP+^low^/CD44- phenotype could be induced. Cells with upregulated MAP2 and Beta III-tubulin expression, and downregulated GFAP expression, express TH (tyrosine hydroxylase), an enzyme required for catecholamines synthesis, which further confirms their neuronal characteristics (Fig. 4a–h). The morphology of these cells, lack of CD44 and high expression of MAP2 and Beta III-tubulin, suggested that they were potential neuronal intermediates (Fig. 5a). Moreover, their percentage was almost as high as the percentage of neuronal cells observed two days later. Among cells showing GFAP and MAP2 co-expression, most (about 70%) presented only the remnants of GFAP, which constitutes additional evidence that the neuronal cells originated from the cells initially showing a multilineage phenotype. The existence of MAP2+, GFAP+, CD44- cells has already been presented by our group [7]. We showed that the percentage of those cells after 5–7 days of neural differentiation did not exceed 5% [7].

### Glioblastoma cells released from the aggregates show features of neural differentiation and differentiation arrest

When cultured under serum-starvation conditions, in contrast to GFAP+NNP, glioblastoma cells formed stable aggregates, and aggregate cells maintained the multilineage phenotype for several months (as currently observed for 8 months). The differences between aggregates of glioblastomas and GFAP+NNP cultures are shown in Fig. 3a,b. When culture conditions were changed to neural differentiation conditions after 12–24 hours, tumor cells migrated from aggregates and formed a monolayer (Fig. 1d,e). After their appearance in monolayer, we continued with two parallel cultures. The monolayer of cells was left in culture for further propagation, and the aggregates were transferred to new dishes.

The monolayer cells released from glioblastoma aggregates presented the multilineage phenotype. In these cells a co-expression of CD44, GFAP, and MAP2 was observed after 24 hours and remained after 5 days of exposure to neural differentiation medium (Fig. 1b,c,d,e). In comparison, after five days of culture in the neural differentiation medium, nearly all GFAP+NNP differentiated into neuronal and astrocytic cells (Fig. 5a,b,c).

After 5–20 days of culture in the neural differentiation medium, the phenotype of the cells released from glioblastoma aggregates was characterized by immunocytochemistry. The results revealed differences in the differentiation patterns and the capacity between cells obtained from examined tumors. GBM2 cells released from aggregates showed the highest resistance to neural differentiation conditions: predominantly, cells sustaining the multilineage phenotype (CD44+/GFAP+/MAP2+) were observed (Table 3; Fig. 5d). GBM1 and GBM3 cells released from aggregates, in the same culture conditions, showed three phenotypes: multilineage (CD44+/GFAP+/MAP2+), non-neural (CD44+/GFAP-/MAP2-) and neuronal intermediate (MAP2+^high^/GFAP+/CD44-) (Table 3; Fig. 5e,f). Comparative analysis showed that the neuronal intermediate phenotype was also observed as a result of GFAP+NNP differentiation (Fig. 5a). However, in GFAP+NNP this phenotype was observed only temporarily prior to differentiation into neural cells, whereas it was very stable in glioblastoma cells. These results suggest that it was possible to trigger the neural phenotype in GBM1 and GBM3 cells, although it was arrested at early stages of neural differentiation. GBM4 cells released from aggregates contained a higher percentage of cells with glial phenotype (greater than 25%) after neural differentiation than GBM1 and GBM3 (Table 3), whereas GBM8 cells contained greater than 40% of glial cells (Table 3; Fig. 5g). In addition to glial, neural differentiation of GBM8 cells yielded a subpopulation resembling neuronal cells (Fig. 5g). In all of these cases (GBM1, GBM2, GBM3, GBM4, GBM8), during the prolonged neural differentiation (15 days), Nestin expression was not eliminated as it was in neuronal derivatives of GFAP+NNP (Fig. 5h) [5]. In parallel, the aggregates were cultured and the phenotype was monitored. After 3–4 months, the aggregates cultured in neural differentiation medium lost the ability to attach to tissue culture surface and to release cells into a monolayer. However, the cells within the aggregates remained viable and displayed the multilineage phenotype (data not shown). These results suggest that in contrast to GFAP+NNP, induction of neural phenotype in the glioblastoma aggregate-derived cell was significantly inhibited.

**Table 3:** Features of neural and mesenchymal differentiation presented by glioblastoma cells

fg	GBM1	GBM2	GBM3	GBM4	GBM8	GFAP+NNP p0	GFAP+NNP p1
Aggregated GBM cells with multilineage phenotype	100	100	100	100	100	no	no

5d Multilineage	100	100	95–100	100	83–87	0–1	0–1

5d NI	0	0	0–2	0	3–6	1–2	1–2

5dMES	0	0	0–2	0	3–5	0	5–8

5dGLIA	0	0	0–3	0	6–10	44–46	45–49

5dNEURO	0	0	0	0	0–3	54–56	47–50

5d not classified	0	0	0–2	0	3–5	0–1	0–1

**20d Multilineage**	**19–23**	**72–75**	**12–15**	**42–46**	**18–22**	**0**	**0**

**20dNI**	**21–25**	**8–12**	**23–25**	**11–15**	**16–20**	**0**	**0**

**20dMES**	**26–29**	**3–6**	**36–40**	**7–11**	**4–6**	**0**	**5–8**

**20dGLIA**	21–24*	4–9*	15–19*	25–31*	44–51*	48–50*	45–44*

**20dNEURO**	**0**	**0**	**0**	**0**	5–7*	50–52*	48–50*

**20d not classified**	**9–12**	**8–11**	**8–13**	**9–12**	**9–11**	**0–1**	**0–1**

GBM, glioblastoma;

GFAP+NNP, normal neural progenitors;

NI, neuronal intermediates;

MES, mesenchymal cells;

GLIA, glial cells;

NEURO, neuronal cells;

5d, five days of differentiation (DMEM/F12 N2);

20d, twenty days of differentiation (DMEM/F12 N2);

no, GFAP+NNP did not show stable aggregates.

*After 5 and 20 days of exposure to the differentiation medium glioblastoma cells showed Nestin expression. GFAP+NNP derivatives showed Nestin expression after 5 but not after 20 days of serum starvation.

### Characteristics of mesenchymal differentiation of glioblastoma cells

**Figure 6:**
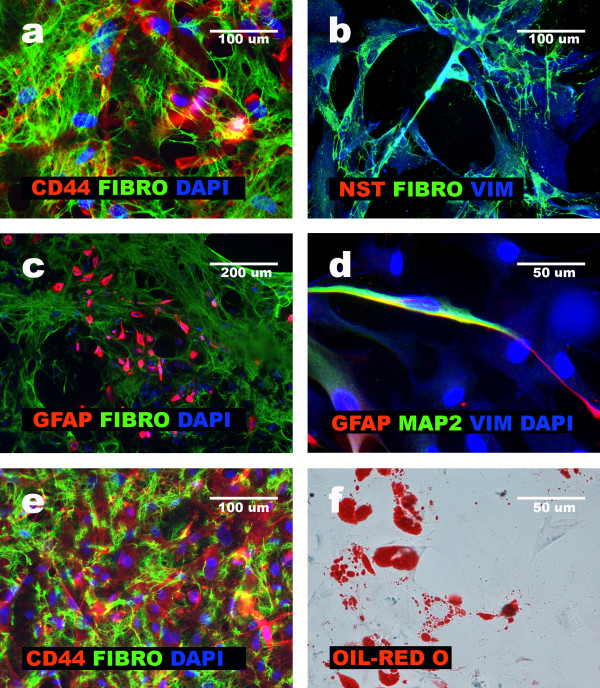
**GFAP+NNP and GBM cells mesenchymal differentiation**. **a**, GFAP+NNP cells positive for CD44 and Fibronectin (FIBRO); **b**, GFAP+NNP cells pasage 5 negative for Nestin (NST) and positive for Vimenitn (VIM) and Fibronectin (FIBRO); **c**, original population of GBM2 cells; population positive for GFAP and Fibronectin and population negative for GFAP but positive for Fibronectin are visible; **d**, GBM4 cells eliminating GFAP and MAP2 but sustaining Vimentin expression; **e**, Expression of CD44 and Fibronectin presented by GBM3 cells at passage12; **f**, cellular derivatives obtained after exposure of GBMs cells to adipogenic differentiation medium.

To observe the mesenchymal differentiation of GFAP+NNP and glioblastoma aggregate-derived cells, the cells were transferred to the alpha-MEM medium supplemented with 10% FBS. When cultured under these conditions, GFAP+NNP generated a population of cells with robust expression of Fibronectin and maintenance of CD44 expression (Fig. 6a,b). We previously defined this population as mesenchymal [8]. We show that similarly to GFAP+NNP, the aggregate-derived cells from GBM1, GBM2, GBM3, GBM4 and GBM8 exposed to alpha-MEM with 10% FBS for a number of passages, were induced to non-neural, mesenchymal phenotype (CD44+/GFAP-/MAP2-) and robustly expressed Fibronectin (Fig. 6c,d). The same effect was observed when heterogeneous glioblastoma cells isolated from GBM1, GBM2, GBM3, GBM4, GBM5 and GBM8, were exposed to alpha-MEM with 10% FBS (Fig. 6e). However, GBM2 and GBM4 cells required more passages (above 15) than GBM1, GBM3, GBM5, GBM8 (above 10), to present purely non-neural, mesenchymal phenotype. Interestingly, in GBM1 and GBM3 aggregate-derived cells, non-neural differentiation was also observed after exposure to neural differentiation medium, as described in the previous paragraph (Fig. 6d). Moreover, glioblastoma-derived mesenchymal cells exposed to adipogenic medium, showed features of adipogenesis (Fig. 6f). Considering our data, and recent literature demonstrating mesenchymal differentiation of glioblastoma cells [1], we defined the CD44+, GFAP-, MAP2- population of glioblastoma cells as mesenchymal.

These results show that two of the analyzed glioblastomas (GBM1 and GBM3) exhibited non-neural differentiation under both neural- and non-neural differentiation conditions.

Molecular analyses (LOH and/or *P53 *sequencing) confirmed that the population of cells with non-neural phenotype consisted entirely of glioblastoma cells and were not contaminated with normal cells as LOH, without contamination with the normal allele, nor were *P53 *mutations identified (Fig. 2c,d).

In addition, our results show that one out of eight glioblastomas (GBM7) did not form aggregates and did not differentiate in accordance with the GFAP+NNP differentiation model (Table 2).

### Glioblastoma cells acquiring mesenchymal features proliferate slowly and do not express CD133

**Figure 7:**
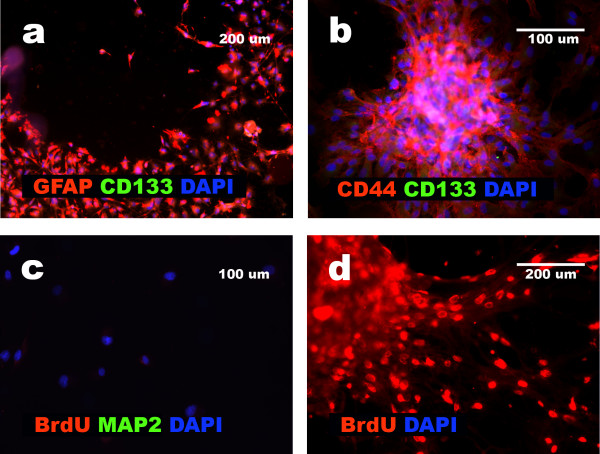
**CD133 expression and BrdU incorporation presented after differentiation of GBMs cells**. **a**, lack of CD133 positive cells amongst cells released from GBM 3 aggregate; **b**, aggregated cells of GBM1 negative for CD133 and positive for CD44; **c**, lack of BrdU incorporation after mesenchymal differentiation of MAP2 negative GBM5 cells; **d**, BrdU incorporation in aggregated and released from aggregate cells of GBM4.

CD133 positive cells were observed in the primary cultures isolated from six out of eight glioblastomas (Table 2; Fig. 1f). All these glioblastomas after exposure as a monolayer culture to the alpha-MEM with 10% FBS, showed a complete loss of CD133-positive cells. Both aggregates of glioblastoma cells and the cells released from the aggregates were negative for CD133 (Fig. 7a,b). Assay of BrdU incorporation revealed that acquisition of the mesenchymal phenotype correlated with very low mitotic activity when compared to the original population of glioblastoma cells isolated from GBM1, GBM 3, GBM 4, GBM 5, GBM 6, and GBM 8 (Fig. 7c,d). Only GBM2 did not show this regression of mitotic activity.

### Majority of glioblastomas do not expand in single cell assay conditions

**Figure 8:**
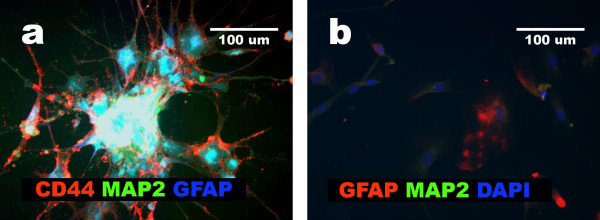
**Single cell assay of aggregated glioblastoma cells**. **a**, GBM2 cells positive for GFAP, MAP2 and CD44; **b**, GBM4: predominant cells negative for GFAP and MAP2; one cell presenting remnants of GFAP is visible.

Glioblastoma cells with the multilineage phenotype obtained from the aggregates and the dispersed cells of all eight tumors were grown in cloning conditions in different media (see Methods). We were able to clone only cells isolated from GBM2, GBM4, and GBM6. GBM2 cells maintained the multilineage phenotype when exposed to serum-starvation medium (Fig. 8a). However, GBM4 and GBM6 acquired mesenchymal phenotype in all tested single cell assay conditions (Fig. 8b). These results showed that single cell assay can be applied only for some glioblastomas. Moreover, single cell assay altered the cellular phenotype in two out of three cloned cell lines. LOH and/or MSI analysis confirmed the neoplastic origin of the cloned cells.

## Discussion

Analysis of the biological potential of glioblastoma cells is important for better understanding of the natural course of the disease. Understanding of the differentiation capacity of glioblastoma cells creates an opportunity of interfering with the glioblastoma phenotype that may appear helpful in the design of new therapies for glioblastomas.

Here we present an *in vitro *model system allowing for: 1) the isolation of glioblastoma cells with multilineage phenotype, 2) a comparison between inducible differentiation of normal neural precursors with multilineage phenotype and cells isolated from human glioblastomas.

Using the *in vitro *model, we have identified the presence of a population of tumor cells with multilineage phenotype among the primary cells obtained from eight examined human glioblastomas. These cells could be isolated from the bulk primary glioblastoma cultures by aggregation of glioblastoma cells with multilineage phenotype. A fraction of glioblastoma cells with multilineage phenotype varied among examined tumors and represented between 10% and 95% of primary tumor cell population (Table 2). In addition, we demonstrated that these tumor cells differentiated, at least to some degree, into cells with neuronal, glial, and mesenchymal phenotypes.

We have previously shown that uncommitted GFAP+NNP co-expressed glial, neuronal and mesenchymal lineage markers, and were capable of multipotent differentiation. After differentiation into glial lineage, neuronal markers were lost, while cells that differentiated into neuronal lineage, lost glial markers [5,6]. We showed also that GFAP+NNP cells differentiated into the mesenchymal lineage, followed by elimination of both neuronal and glial markers [7]. Similar observations were reported before by Egusa et al. and Zipori et al. who described undifferentiated cells with a "discordant phenotype", which means simultaneous expression of markers characteristic for different lineages by the same cell, defining these cells as stem cells [14,15]. While differentiating, these cells gradually "silence" superfluous genes and acquire a specific phenotype by up-regulation of specific markers [14,15]. Thus, the process of differentiation is perceived as a complex molecular mechanism that ultimately "switches off" expression of superfluous genes and articulates expression of genes required for a final phenotype. That model may explain a phenomenon of simultaneous expression of neuronal, glial and mesenchymal markers in glioblastoma cells that are able to differentiate into different phenotypes [1,2].

In our culture model, a population of glioblastoma cells was able to form aggregates composed of cells with multilineage phenotype, i.e. showing expression of: CD44, Vimentin, GFAP, Nestin, Beta III-tubulin, MAP2, Fibronectin and SOX-2, but not CD133. This phenotype was shared by GFAP+NNP before differentiation [5-7]. This is a very interesting observation in light of recent reports demonstrating that not only CD133 positive cells, but also CD133 negative cells, are capable of tumor initiation [16]. SOX2 is marker of neural stem cells/progenitors [17].

According to our data, up to 95% of tumor cells isolated from human glioblastomas e.g. GBM1 show co-expression of glial, neuronal and mesenchymal markers. However, in contrast to GFAP+NNP, glioblastoma cells with multilineage phenotype appeared to be very resistant to our neural differentiation medium. One discrepancy between glioblastoma cells and GFAP+NNP was sustained Nestin expression in neural derivatives of glioblastoma cells. Nestin is a marker of undifferentiated neuroectodermal cells [18]. Although, glioblastoma cells showed some features of neural and/or mesenchymal differentiation after exposure to differentiation medium, the time and culture conditions required for the differentiation, and the final phenotype, differed among all examined tumors. For example, GBM2-derived cells sustained the multilineage phenotype after serum-starvation, whereas GBM1 tumor cells differentiated efficiently to mesenchymal cells in any of the differentiation conditions used in this study. GBM8 showed more advanced neuronal differentiation, whereas GBM1, GBM3 and GBM4 showed an ability to produce neuronal intermediates only. Although the advanced neural differentiation was inhibited in a majority of glioblastomas, at least the intermediate neuronal phenotype was effectively induced in glioblastoma cells. We suggest that the MAP2+^HIGH^/GFAP+^LOW^/CD44- phenotype, previously described by us [7], represents the intermediate neuronal phenotype observed shortly after the onset of GFAP+NNP neural differentiation. These results, together with the multilineage phenotype of uncommitted GFAP+NNP, suggest that a continuation of multilineage phenotype in glioblastoma cells is a consequence of differentiation arrest.

Our data support previous observations that glioblastoma represents a neoplasm capable of a vast phenotypic diversity in differentiation patterns [1,3,16]. Interestingly, after induction of differentiation one common phenotype, mesenchymal, was shared by six of the eight glioblastomas analyzed in this study. The mesenchymal phenotype associated with glioblastoma was previously described by Tso et al. [3]. We propose that the mesenchymal differentiation of GBM cells can be recognized as a characteristic of cells with multilineage phenotype e.g. GFAP+NNP.

The previously published results showing common origin of gliomatous and sarcomatous component in gliosarcomas, [19,20], and recently presented, *in vitro *observations of Tso et al. and Ricci-Vitiani et al. point to the common origin of glioblastoma [3,4]. However, the cellular origin of glioblastoma is not known. Considering the facts presented above, a population of glioblastoma cells identified in this study resembles GFAP+NNP in terms of their multilineage phenotype before differentiation, and the capacity, at least to some degree, for neural and mesenchymal differentiation. Tso et al. proposed two hypotheses explaining GBM origin. One states that a subset of primary glioblastomas may be derived from transformed stem cells containing Mesenchymal Stem Cells (MSC)-like properties and retain partial phenotypic aspects of MSC nature in tumors. According to the second hypothesis, glioblastomas activate a series of genes that result in mesenchymal properties of the cancer cells that sustained tumor growth and malignant progression [3]. We support the first Tso et al. proposal, and we suggest that cells containing mesenchymal-like characteristics, like GFAP+NNP, can be transformed into glioblastoma cells [3]. Based on the observed mesenchymal differentiation of glioblastoma cells, Ricci-Vitiani et al. proposed that in a subclass of glioblastomas, the transformation hit occurs in a multipotent stem cell, which may reveal its plasticity under specific environmental stimuli [4]. It is possible that, a population of multipotent progenitor cells with neural and mesenchymal characteristics, such as GFAP+NNP, represent this kind of plasticity.

An example of phenotypic plasticity *in vitro and in vivo *is the development of mesenchymal cells from neural crest [21]. It was shown previously that neural stem cells have the ability to differentiate into neural crest cells [22], and may represent this kind of plasticity. Our recent studies showed that GFAP+NNP, in addition to neural and mesenchymal, also have characteristics of neural crest [8]. In this scenario perhaps "neural crest-like to mesenchymal transition", instead of mesenchymal differentiation of glioblastoma cells, would be more appropriate to convey the connection between glioblastoma cells and multipotent GFAP+NNP.

After induction into the mesenchymal phenotype, glioblastoma cultures lost CD133 population and their proliferation rate substantially decreased. These results suggest that extrinsic factors responsible for an efficient mesenchymal differentiation should be investigated more thoroughly, since acquisition of mesenchymal differentiation correlates with the characteristics of cell senescence. This approach may provide an opportunity to reduce the aggressive behavior of some neoplasms *in vivo*.

**Figure 9:**
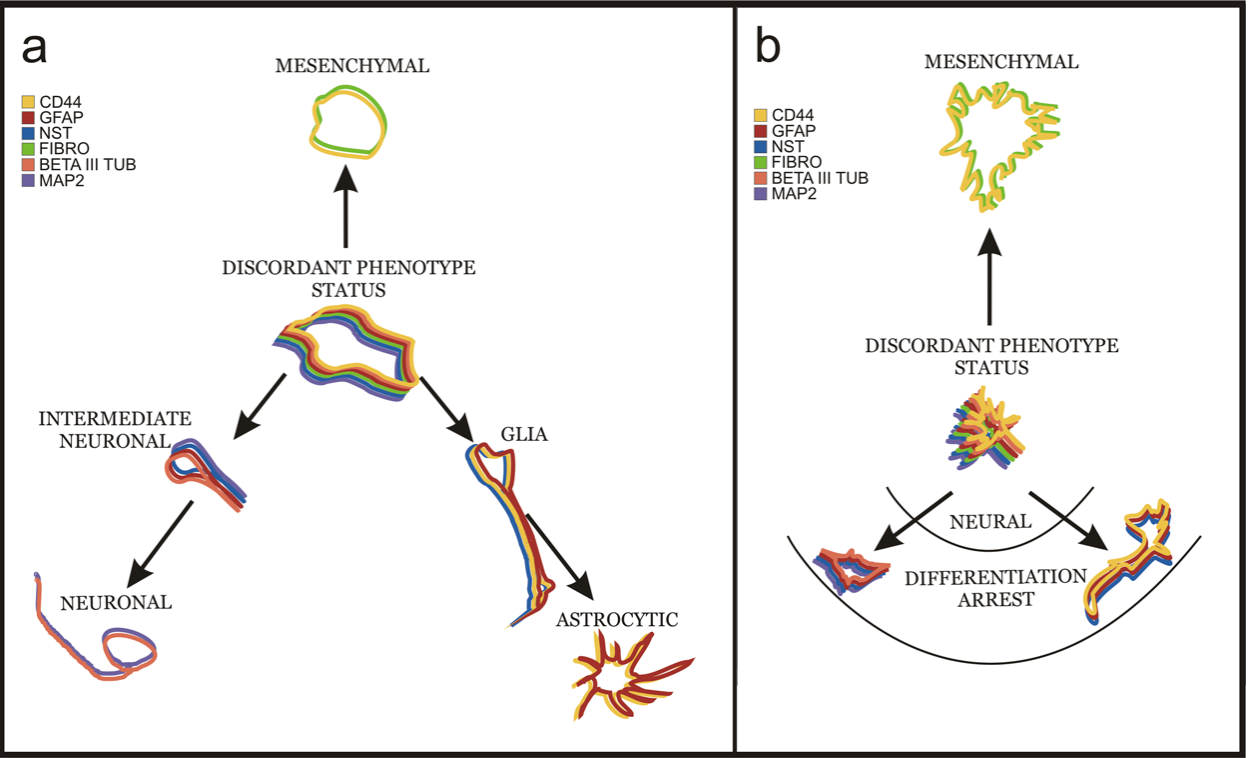
**Stages and pathways of GFAP+NNP and GBM cells differentiation**. **a**, GFAP+NNP cells coexpress Nestin (NST), Fibronectin (FIBRO), GFAP, MAP2, CD44 and Beta III-tubulin. Neural intermediants express GFAP, MAP2, Nestin and Beta III-tubulin. Neuronal cells express MAP2, Beta III-tubulin. Glial cells coexpress GFAP, Nestin and CD44. Non-neural cells express CD44 and Fibronectin. **b**, in GBM cells the mesenchymal differentiation is very advanced; neural differentiation is arrested at early stages.

Considering our results, the co-expression of glial, neuronal and mesenchymal markers in glioma cells cannot be regarded as an anomaly. It has been shown before that undifferentiated cells isolated from different tissues may co-express multilineage markers [23,24]. A co-expression of multilineage markers preceding a commitment in the hematopoietic system was reported by Hu et al. [24]. The multilineage phenotype of cultured glioblastoma cells may represent a stabilized phenotype that is temporarily expressed by normal uncommitted multipotent cells such as GFAP+NNP, and lost after differentiation (Fig. 9). They differentiate in accordance with the so called model of superfluous genes suppression [15], while the capability of acquisition of a more mature phenotype is inhibited to a vast extent in glioblastomas. It should be stressed that according to our data up to 95% glioblastoma cells (GBM1) co-express multilineage markers.

An additional observation also appears from our analysis. Cloning is commonly used as the first step of glioblastoma cells analysis. Here we confirmed that not all glioblastomas can be analysed by this technique [1]. It should be also considered that cloning changes the original phenotype of glioblastoma cells.

## Conclusion

In conclusion, we showed that human glioblastomas contain cells with multilineage phenotype. According to our data up to 95% of glioblastoma cells can present multilineage phenotype. These cells have remarkable phenotypic similarities to neural progenitors and express neural and mesenchymal markers before differentiation. The co-expression of neuronal and glial markers is transient or stable during the differentiation of GFAP+NNP and glioblastoma cells, respectively. Our data suggest that the co-expression of neuronal and glial markers in glioblastoma cells results from a differentiation arrest (Fig. 9). We support the suggestions of Tso et al. and Ricci-Vitiani et al. that a subset of glioblastomas is developed from transformed stem cells, with mesenchymal stem cells properties, or multipotent stem cells, which may reveal their plasticity under specific environmental stimuli [3,4]. Multipotent cells, with mesenchymal characteristics similar to GFAP+NNP, can represent these kinds of cells. Mesenchymal-like differentiation of glioblastoma cells, as well as the previously described mesenchymal differentiation of GFAP+NNP, was environmentally regulated, and mesenchymal differentiation could be induced in majority of glioblastoma cells. The latter phenomenon was associated with cellular senescence and elimination of CD133 positive cells. These data may indicate an alternative therapeutic approach to glioblastoma.

## Abbreviations

BrdU: Bromodeoxyuridine; DMEM: Dulbecco's Minimal Essential Medium; EGFR: epidermal growth factor receptor; FBS: fetal bovine serum; FIBRO: fibronectin; GBM: glioblastoma; GFAP: glial fibrilary acidic protein; GFAP+NNP: GFAP positive normal neural progenitors; IBMX: Isobutylmethylxanthine; MEM: Minimum Essential Medium; NHA: normal human astrocytes; LOH: loss of heterozygosity; MAP2: microtubule associated protein; MSI: microsatellite instability; NST: nestin; PBS: Phosphate buffered saline; rhEGF: recombinant human eidermal growth factor; SDS-PAGE: Sodium Dodecyl Sulfate Polyacrylamide Gel Electrophoresis; SOX-2: SRY-related HMG-box gene 2; VIM: vimentin.

## Competing interests

The authors declare that they have no competing interests.

## Authors' contributions

PR designed project. PR, KH-B, PS, MZ performed cells cultures. PR, MW-P, MZ participated in obtaining immunocytochemistry data. MS and WB performed LOH, multiplex and sequencing analysis. PR and EG were responsible for funding acquisition. DJJ and KZ participated in providing samples for experiments. PPL, KB, WB participated in western blot. MW performed experiments required for revision. All authors participated in analysis and interpretation of obtained data. All authors have been involved in drafting the manuscript. All authors have given approval of the final version of the manuscript.

## Pre-publication history

The pre-publication history for this paper can be accessed here:

http://www.biomedcentral.com/1471-2407/9/54/prepub

## References

[B1] Günther HS Schmidt NO Phillips HS Kemming D Kharbanda S Soriano R Modrusan Z Meissner H Westphal M Lamszus K Glioblastoma-derived stem cell-enriched cultures form distinct subgroups according to molecular and phenotypic criteria Oncogene 2008 27 2897 909 10.1038/sj.onc.1210949 18037961

[B2] Ignatova TN Kukekov VG Laywell ED Suslov ON Vrionis FD Steindler DA Human cortical glial tumors contain neural stem-like cells expressing astroglial and neuronal markers in vitro Glia 2002 39 193 206 10.1002/glia.10094 12203386

[B3] Tso CL Shintaku P Chen J Liu Q Liu J Chen Z Yoshimoto K Mischel PS Cloughesy TF Liau LM Nelson SF Primary glioblastomas express mesenchymal stem-like properties Mol Cancer Res 2006 9 607 19 10.1158/1541-7786.MCR-06-0005 16966431

[B4] Ricci-Vitiani L Pallini R Larocca LM Lombardi DG Signore M Pierconti F Petrucci G Montano N Maira G De Maria R Mesenchymal differentiation of glioblastoma stem cells Cell Death Differ 2008 15 1491 8 10.1038/cdd.2008.72 18497759

[B5] Rieske P Azizi SA Augelli B Gaughan J Krynska B A population of human brain parenchymal cells express markers of glial, neuronal and early neural cells and differentiate into cells of neuronal and glial lineages Eur J Neurosci 2007 25 1 31 37 17241264 10.1111/j.1460-9568.2006.05254.x

[B6] Witusik M Gresner SM Hulas-Bigoszewska K Krynska B Azizi SA Liberski PP Brown P Rieske P Neuronal and astrocytic cells, obtained after differentiation of human neural GFAP-positive progenitors, present heterogeneous expression of PrPc Brain Res 2007 1186 65 73 10.1016/j.brainres.2007.10.039 17996224

[B7] Witusik M Piaskowski S Hulas-Bigoszewska K Zakrzewska M Gresner SM Azizi AS Krynska B Liberski PP Rieske P Successful elimination of non-neural cells and unachievable elimination of glial cells by means of commonly used cell culture manipulations during differentiation of GFAP and SOX2-positive neural progenitors (NHA) to neuronal cells BMC Biotechnol 2008 8 56 18638414 10.1186/1472-6750-8-56 PMC2488339

[B8] Rieske P Augelli BJ Stawski R Gaughan J Azizi SA Krynska B A Population of Human Brain Cells Expressing Phenotypic Markers of More Than One Lineage Can Be Induced In Vitro to Differentiate into Mesenchymal Cells Exp Cell Res 2009 315 462 73 10.1016/j.yexcr.2008.11.004 19061885

[B9] Rieske P Kordek R Bartkowiak J Debiec-Rychter M Biernat W Liberski PP comparative study of epidermal growth factor receptor (EGFR) and MDM2 gene amplification and protein immunoreactivity in human glioblastomas Pol J Pathol 1998 49 145 9 9810172

[B10] Szybka M Bartkowiak J Zakrzewski K Polis L Liberski P Kordek R Microsatellite instability and expression of DNA mismatch repair genes in malignant astrocytic tumors from adult and pediatric patients Clin Neuropathol 2003 22 180 6 12908754

[B11] Ohgaki H Eibl RH Schwab M Reichel MB Mariani L Gehring M Petersen I Höll T Wiestler OD Kleihues P Mutations of the p53 tumor suppressor gene in neoplasms of the human nervous system Mol Carcinog 1993 8 74 80 10.1002/mc.2940080203 8397797

[B12] Biernat W Tohma Y Yonekawa Y Kleihues P Ohgaki H Alterations of cell cycle regulatory genes in primary (de novo) and secondary glioblastomas Acta Neuropathol 1997 94 303 309 10.1007/s004010050711 9341929

[B13] von Deimling A von Ammon K Schoenfeld D Wiestler OD Seizinger BR Louis DN Subsets of glioblastoma multiforme defined by molecular genetic analysis Brain Pathol 1993 3 19 26 10.1111/j.1750-3639.1993.tb00721.x 8269081

[B14] Zipori D The nature of stem cells: state rather than entity Nat Rev Genet 2004 5 873 878 10.1038/nrg1475 15520797

[B15] Egusa H Schweizer FE Wang CC Matsuka Y Nishimura I Neuronal differentiation of bone marrow-derived stromal stem cells involves suppression of discordant phenotypes through gene silencing J Biol Chem 2005 280 23691 23697 10.1074/jbc.M413796200 15855172

[B16] Beier D Hau P Proescholdt M Lohmeier A Wischhusen J Oefner PJ Aigner L Brawanski A Bogdahn U Beier CP CD133(+) and CD133(-) glioblastoma-derived cancer stem cells show differential growth characteristics and molecular profiles Cancer Res 2007 67 4010 4015 10.1158/0008-5472.CAN-06-4180 17483311

[B17] Ellis P Fagan BM Magness ST Hutton S Taranova O Hayashi S McMahon A Rao M Pevny L SOX2, a persistent marker for multipotential neural stem cells derived from embryonic stem cells, the embryo or the adult Dev Neurosci 2004 26 148 165 10.1159/000082134 15711057

[B18] Temple S The development of neural stem cells Nature 2001 414 112 117 10.1038/35102174 11689956

[B19] Biernat W Aguzzi A Sure U Grant JW Kleihues P Hegi ME Identical mutations of the p53 tumor suppressor gene in the gliomatous and the sarcomatous components of gliosarcomas suggest a common origin from glial cells J Neuropathol Exp Neurol 1995 54 651 656 10.1097/00005072-199509000-00006 7666053

[B20] Boerman RH Anderl K Herath J Borell T Johnson N Schaeffer-Klein J Kirchhof A Raap AK Scheithauer BW Jenkins RB The glial and mesenchymal elements of gliosarcomas share similar genetic alterations J Neuropathol Exp Neurol 1996 55 973 81 10.1097/00005072-199609000-00004 8800093

[B21] McBratney-Owen B Iseki S Bamforth SD Olsen BR Morriss-Kay GM Development and tissue origins of the mammalian cranial base Dev Biol 2008 322 121 32 10.1016/j.ydbio.2008.07.016 18680740 PMC2847450

[B22] Sailer MH Hazel TG Panchision DM Hoeppner DJ Schwab ME McKay RD BMP2 and FGF2 cooperate to induce neural-crest-like fates from fetal and adult CNS stem cells J Cell Sci 2005 118 5849 60 10.1242/jcs.02708 16339968

[B23] Laywell ED Kearns SM Zheng T Chen KA Deng J Chen HX Roper SN Steindler DA Neuron-to-astrocyte transition: phenotypic fluidity and the formation of hybrid asterons in differentiating neurospheres J Comp Neurol 2005 493 321 33 16261530 10.1002/cne.20722 PMC2571943

[B24] Hu M Krause D Greaves M Sharkis S Dexter M Heyworth C Enver T Multilineage gene expression precedes commitment in the hemopoietic system Genes Dev 1997 11 774 85 10.1101/gad.11.6.774 9087431

